# Insights into RNAi-based antiviral immunity in Lepidoptera: acute and persistent infections in *Bombyx mori* and *Trichoplusia ni* cell lines

**DOI:** 10.1038/s41598-018-20848-6

**Published:** 2018-02-05

**Authors:** Dulce Santos, Niels Wynant, Stijn Van den Brande, Thomas-Wolf Verdonckt, Lina Mingels, Paulien Peeters, Anna Kolliopoulou, Luc Swevers, Jozef Vanden Broeck

**Affiliations:** 1 Research group of Molecular Developmental Physiology and Signal Transduction, KU Leuven, Naamsestraat 59, box 02465, 3000 Leuven, Belgium; 2 Insect Molecular Genetics and Biotechnology Group, Institute of Biosciences and Applications, National Center for Scientific Research “Demokritos”, 153 10 Aghia Paraskevi Attikis, Athens, Greece

## Abstract

The control of viral infections in insects is a current issue of major concern and RNA interference (RNAi) is considered the main antiviral immune response in this group of animals. Here we demonstrate that overexpression of key RNAi factors can help to protect insect cells against viral infections. In particular, we show that overexpression of Dicer2 and Argonaute2 in lepidopteran cells leads to improved defense against the acute infection of the Cricket Paralysis Virus (CrPV). We also demonstrate an important role of RNAi in the control of persistent viral infections, as the one caused by the Macula-like Latent Virus (MLV). Specifically, a direct interaction between Argonaute2 and virus-specific small RNAs is shown. Yet, while knocking down Dicer2 and Argonaute2 resulted in higher transcript levels of the persistently infecting MLV in the lepidopteran cells under investigation, overexpression of these proteins could not further reduce these levels. Taken together, our data provide deep insight into the RNAi-based interactions between insects and their viruses. In addition, our results suggest the potential use of an RNAi gain-of-function approach as an alternative strategy to obtain reduced viral-induced mortality in Lepidoptera, an insect order that encompasses multiple species of relevant economic value.

## Introduction

RNA interference (RNAi) is a post-transcriptional gene-silencing mechanism directed by small RNA molecules. According to their structure and biogenesis, these molecules are classified in three main groups: micro (mi)RNAs, PIWI-interacting (pi)RNAs and small interfering (si)RNAs. This classification is further supported by the distinct biological processes in which the different small RNAs are involved: miRNAs are important effectors of endogenous gene regulation, piRNAs are essential defenders of genomic integrity by controlling retrotransposon activity and siRNAs are crucial antiviral effectors. Although crosstalk between the different RNAi pathways and their functions has been described, the siRNA-directed RNAi (siRNAi) is consensually considered the most important antiviral response in insects^1,2^.

Three major lines of evidence demonstrate the importance of this pathway for insect antiviral immunity. First, loss-of-function studies showed that impairment of siRNAi leads to a compromised antiviral immune response, both at intracellular and systemic levels^3,4^. Second, numerous deep sequencing studies have identified and characterized virus-derived siRNAs in insects^2^. Third, viruses have evolved mechanisms to counteract the effectiveness of this pathway, for instance through the expression of Viral Suppressors of RNAi (VSRs)^2,3,5^. Furthermore, key components of the siRNAi pathway have been demonstrated to be among the fastest evolving genes in *Drosophila*, which strongly supports a role in (antiviral) immunity^6,7^. Thus, although other immune pathways have been reported to be involved during specific viral infections (e.g. Toll, Imd and JAK-STAT, among others), siRNAi is considered the most efficient and broadly–acting antiviral response in insects^2,8,9^.

The siRNAi pathway is activated when long dsRNA molecules, mainly produced during the replicative cycle of viruses, are recognized and diced into siRNAs (18–24 bp) by the RNase type III enzyme Dicer2 (Dcr2). These siRNAs are then incorporated into an RNA-Induced Silencing Complex (RISC), where they are unwound, making the guide strand available to direct target RNA recognition by Watson-Crick base pairing. At this point, the catalytic component of RISC, Argonaute2 (Ago2), mediates the degradation of complementary viral RNA, thus combating the viral infection^1^. For this reason, dsRNA is an important pathogen associated molecular pattern (PAMP) for viral infection, triggering a robust degradation of viral RNA via siRNAi^10,11^.

Viral infections can be generally classified in two types, namely acute and persistent. Acute infections are characterized by high levels of viral replication and viral particle production. Generally, these infections are limited in time either by the death of the host or by the clearance of the virus by the host’s immune system. On the other hand, persistent infections typically result in lower viral replication levels, as well as lower production of viral particles. These infections can last for a long time due to an established equilibrium between the attack and counterattack strategies of the virus-host system. In addition, although some persistent infections have the potential to cause variable levels of sublethal effects, it is also frequent that no obvious symptoms are observed^5,12–14^. Therefore, since persistent viral infections do not always cause obvious pathogenesis, their existence is often neglected. However, due to the advance of genomic and transcriptomic tools, identification of persistent viruses has become more recurring, with several reported cases both *in vivo* and in cultured cells^15–20^. In this context, it is noteworthy that, although most insect RNAi-based antiviral immunity research has been performed in models of acute infection, it has also been demonstrated that the siRNAi machinery is involved in the defence against persistent infections in *Drosophila* cells^21^. Nevertheless, the mechanisms underlying persistent viral infections in other insects remain to be elucidated. A clear example of this refers to the economically important order of Lepidoptera, with several persistent viral infections well reported^5,19,20,22^.

Interestingly, while siRNAi is naturally activated as a defense mechanism against viral infections, this pathway can also be triggered by artificial delivery of gene-specific long dsRNA, leading to specific endogenous gene silencing^1^. This RNAi-based technology has important applications in several fields, such as in functional genomics research for studying knockdown phenotypes or in pest control for the development of novel, highly specific insecticides^23–28^. Yet, the successful use of the RNAi technology is highly insect species-dependent, with certain species and orders known to be particularly more insensitive to artificially delivered dsRNA than others. Many insects from the order of Lepidoptera are good examples of such insensitivity^1,5,13^. In this context, it has been demonstrated that overexpression of key siRNAi factors leads to an enhanced silencing response upon delivery of gene-specific dsRNA in the important lepidopteran model organism, *Bombyx mori*^29,30^. Therefore, considering the natural crucial role of the siRNAi pathway in circumventing viral infections, we decided to investigate whether an enhanced siRNAi response would result in an improved antiviral response in lepidopteran cells.

Improvement of antiviral immunity in insects is of particular interest. On one hand, different mosquito species are central vectors for human viral diseases^9^. On the other hand, species of high ecological and economic value, such as silk moths and bees, are highly affected by viral diseases that culminate in relevant losses. Importantly, genetically engineered silkworms expressing (several) dsRNA(s) specific to the *B. mori* Nuclear Polyhedrosis Virus (BmNPV) have been obtained, which resulted in enhanced resistance to this harmful infection^31–33^. In fact, this approach has already been successfully applied to a commercial and highly valuable silkworm strain^33^. Moreover, delivery of viral-specific dsRNA has been considered for protection of honey bees against acute viral infections^34,35^.

In order to investigate the possibility of improving antiviral immunity in Lepidoptera, particularly in lepidopteran cell lines, via overexpression of the siRNAi-machinery, we selected two well-known insect viruses, namely the Cricket Paralysis Virus (CrPV) and the Macula-like Latent Virus (MLV). These are positive single-stranded RNA viruses that generally cause infections with different levels of virulence. On one hand, CrPV causes acute infections in a wide range of insect hosts^36^. On the other hand, MLV is well known to generate persistent infections in lepidopterans^5,22,37^. In addition, CrPV encodes a well-described VSR, named 1 A, which directly interferes with the Ago2 function; while, to our knowledge, no MLV-VSR has been identified as yet^5,22,38^.

In this paper, we report a stronger antiviral response in High Five cells overexpressing key RNAi components, namely Dicer2 and Argonaute2, during an acute viral infection with CrPV. Interestingly, a similar approach does not result in decreased transcript levels of the persistently infecting MLV. Nevertheless, our results indicate that the siRNAi pathway plays a role in the control of persistent viral infections in lepidopteran cell lines.

## Results

### Overexpression of Dcr2 and Ago2 leads to a reduced CrPV-induced mortality

**Figure 1:**
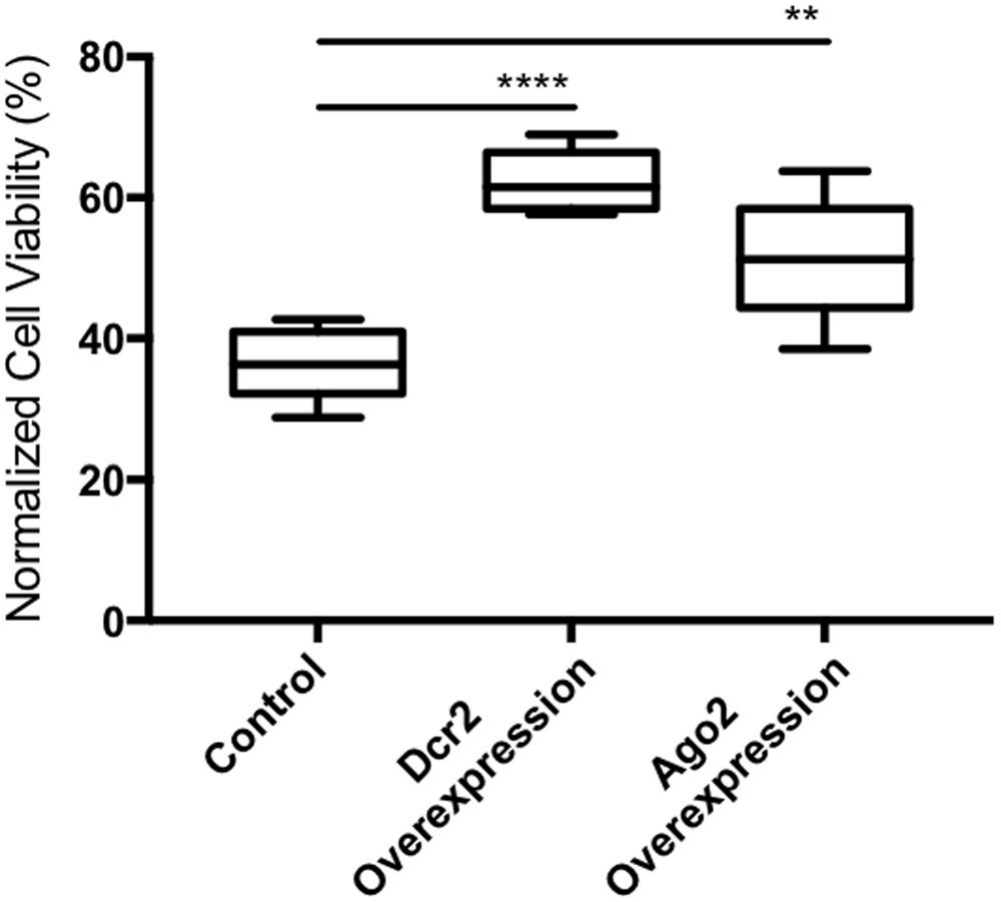
Overexpression of Dcr2 and Ago2 results in improved viability upon CrPV infection in High Five cells. Cells were transfected with overexpression constructs containing the entire ORF of *Bm*-*dcr2* and *Bm*-*ago2*. A group transfected with the pEA-pac control vector was used as control. The transfected cells were infected with CrPV (MOI25), or treated with PBS, and the viability was assessed after 48 h. The graph depicts box-plots of the normalized cell viability, i.e. the viability of the CrPV-infected cells normalized to the viability of the PBS-treated cells, in percentage. Statistical analysis (Shapiro-Wilk normality test and Unpaired T-tests) was performed in GraphPad Prism 7 (****P < 0.0001; **P < 0.01; n = 6).

The first main objective of this study consists of investigating whether siRNAi-machinery overexpression results in an enhanced response against acute viral infections. This is of particular interest since overexpression of Dcr2 or Ago2 leads to an improved gene-silencing response in lepidopteran cells^29^. We thus worked with two important lepidopteran cell lines, namely the *Trichoplusia ni* High Five cells and the *B. mori* Bm5 cells, and with the well-characterized Cricket Paralysis Virus (CrPV). High Five cells were shown to be more sensitive to CrPV infection and were thereby selected to continue the study (Fig. S1, supplementary data). We then tested whether overexpression of Dcr2 or Ago2 resulted in improved antiviral response in the previously selected High Five cells. Overexpression of these proteins was analyzed via Western blot and clearly proved to occur in High Five cells. This was compared with the control group, for which an equal amount of total protein was loaded but no signal of Dcr2 or Ago2 was detected (Fig. S2, supplementary data). Importantly, overexpression of Dcr2 or Ago2 resulted in reduced virus-induced mortality upon CrPV infection, in comparison with control cells (Fig. 1).

**Figure 2:**
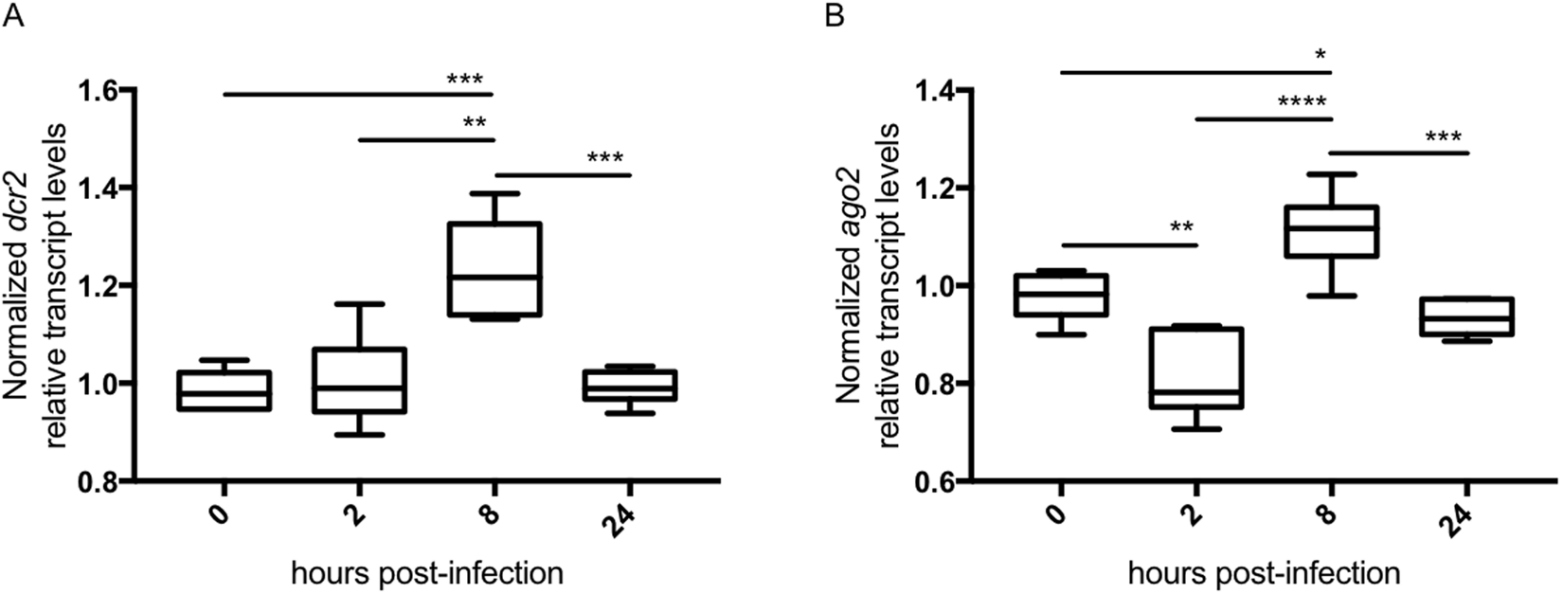
Differential regulation of *dcr*2 and *ago2* upon CrPV infection in High Five cells. Cells were infected with CrPV (MOI10) or treated with PBS. The transcript levels of *Tn-dcr2* (**A**) and *Tn-ago2* (**B**) were measured 0, 2, 8 and 24 h post-infection. The graphs depict box-plots of the normalized relative transcript levels, i.e. the relative transcript levels of the CrPV-infected cells normalized to the relative transcript levels of the PBS-treated cells. Statistical analysis (Shapiro-Wilk normality test and Unpaired T-tests) was performed in GraphPad Prism 7 (*P < 0.05; **P < 0.01; ***P < 0.005; ****P < 0.0001; n = 4).

Next, we tested whether the endogenous siRNAi components, namely *T. ni* Dcr2 and Ago2, are differentially regulated in High Five cells upon infection with CrPV. In order to quantify the levels of *T. ni dcr2* and *ago2*, the sequences of these transcripts were first identified from the publically available transcriptome of High Five cells^39^ and further confirmed via protein domain prediction and phylogenetic analysis (Fig. S3, supplementary data). In addition, an alignment of the amino-acid sequences of *T. ni* and *B. mori* Dcr2 and Ago2 was performed (Fig. S4, supplementary data). Next, we infected High Five cells with CrPV and quantified the *dcr2* and *ago2* transcript levels in course of time. Differential regulation of both transcripts was observed. Mild upregulations were detected 8 hours post-infection (h.p.i.), which stabilized at 24 h.p.i. (Fig. 2). Interestingly, a small downregulation of *ago2* was observed at 2 h.p.i. (Fig. 2B).

### Overexpression of Dcr2 and Ago2 does not result in decreased transcript levels of the Macula-like Latent Virus (MLV)

**Figure 3:**
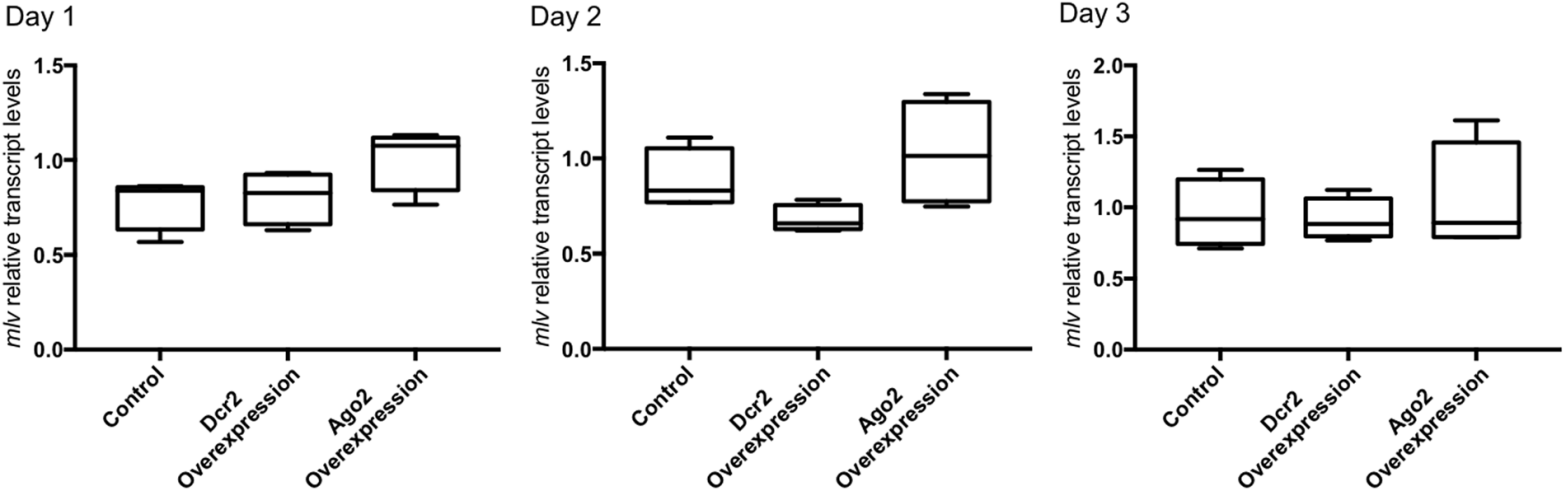
Overexpression of Dcr2 and Ago2 does not induce changes in the *mlv* transcript levels in High Five cells. Cells were transfected with overexpression constructs containing the entire ORF of *Bm*-*dcr2* and *Bm*-*ago2*. A group transfected with the pEA-pac control vector was used as control. The *mlv* relative transcript levels were assessed 1, 2 and 3 days after the transfection, respectively. The graphs depict box-plots of *mlv* relative transcript levels. Statistical analysis (Shapiro-Wilk normality test and Unpaired T-tests) was performed in GraphPad Prism 7 (n = 4).

Since overexpression of Dcr2 and Ago2 resulted in an improved survivorship to the acute infection of CrPV, we checked whether this approach would result in decreased levels of a persistent viral infection. A Macula-like Latent Virus (MLV) has been identified in *B. mori* cultured cells and its presence has been identified in High Five cell stocks, without any apparent pathogenic effects^5,22^. In line with these reports, an MLV infection also occurs in the High Five cells available in our lab (Fig. S5, supplementary data). Since MLV-induced mortality is not observed, *mlv* transcript levels were measured instead. Yet, upon overexpression of Dcr2 and Ago2, statistically significant changes could not be observed in any of the time-points (one, two and three days after transfection) (Fig. 3).

### Virus-specific small interfering (si)RNAs are bound to Ago2

**Figure 4:**
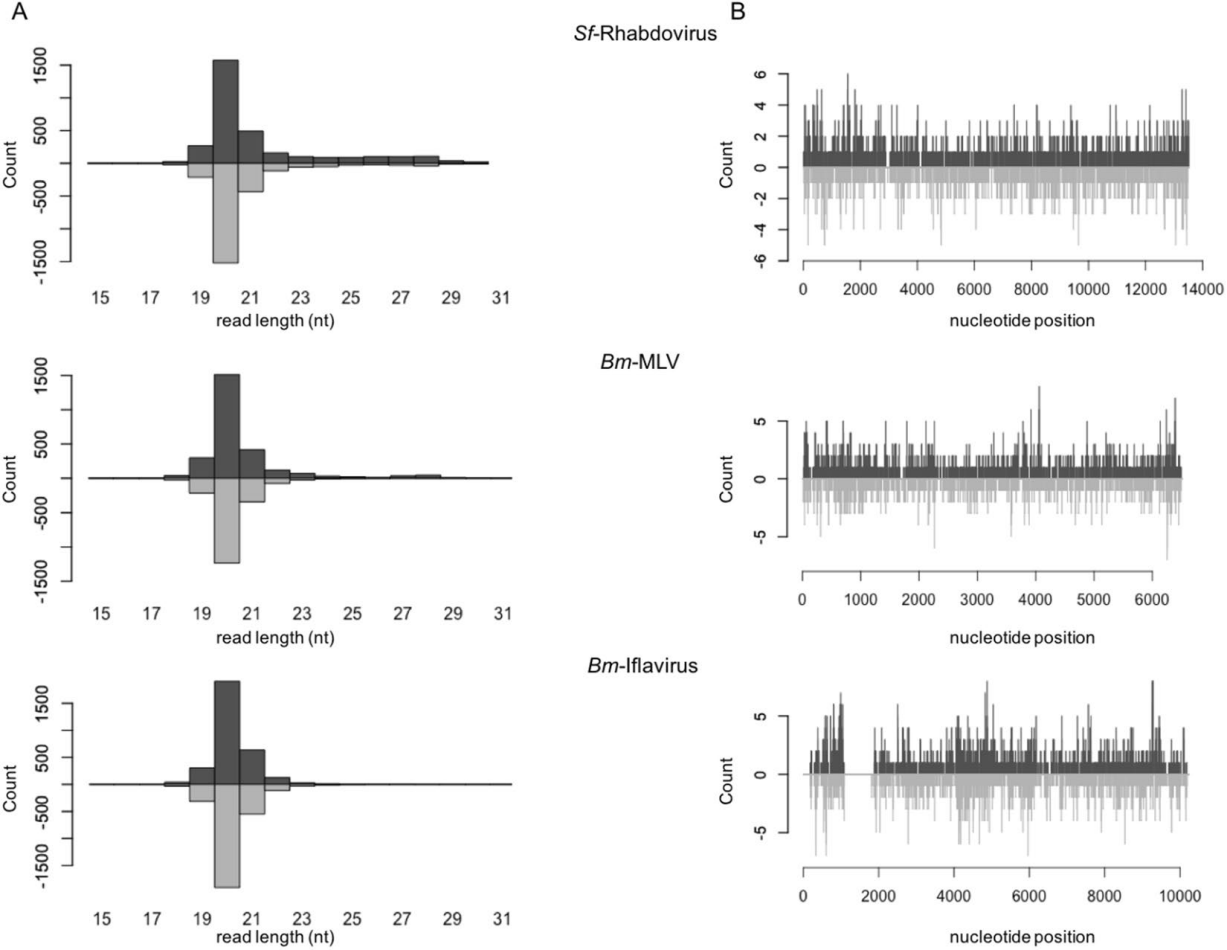
Virus-specific siRNAs are bound to Ago2 in BmN cells. (**A**) Size distribution of the Ago2-bound viral reads (*Sf*-RV, *Bm*-MLV and *Bm*-IV), (**B**) Ago2-bound siRNAs map to the genome of *Sf*-RV, *Bm*-MLV and *Bm*-IV. (black: sense reads; grey: anti-sense reads).

Although overexpression of Dcr2 and Ago2 did not induce obvious changes in the *mlv* transcript levels, the question whether virus-specific siRNAs can directly interact with the RNAi machinery to eventually play a role in the defense against persistent viral infections remains unanswered. Therefore, in order to study this matter, and based on the methodology described by Wu and colleagues^17^, we investigated if potential viral genomes could be retrieved upon assembly of small RNAs bound to Ago2 in *B. mori* BmN cells. This cell line was selected since a BmN-derived library of deep sequenced small RNAs obtained by Ago2 immunoprecipitation and RNA sequencing (RIPseq) is publically available^40^. For the analysis of this library (SRX201604, GMS1025527_Unannotated_smal_RNAs), the reads that mapped to the *B. mori* genome (ASM15162v1, GCA_000151625.1) were removed, so that only the unmapped reads were assembled into contigs. These contigs were subsequently used to perform a BLAST search against a database of viral genomes, which resulted in the identification of three viral genomes that have been previously reported to maintain persistent infections in lepidopterans: (i) *Spodoptera frugiperda* Rhabdovirus (*Sf*-RV), which is a (−)ssRNA genome virus that has been identified for the first time in Sf9 cells^19^, (ii) the *B. mori* Iflavirus (*Bm*-IV1), which is a (+)ssRNA that has been previously discovered to be persistently present in *Bombyx* pupa^20^ and (iii) MLV. The size distribution of the virus-specific reads revealed a peak at 20 bp, which is a typical characteristic of siRNAs (Fig. 4A). A direct mapping of size-selected reads (18–24 bp) on the viral genomes demonstrated that the siRNAs evenly map on the viral genomes (Fig. 4B). Interestingly, siRNAs mapping to a small region of the *Bm*-IV1 were absent (Fig. 4B).

### Knockdowns of *dcr2* and *ago2* result in increased *mlv* transcript levels

**Figure 5:**
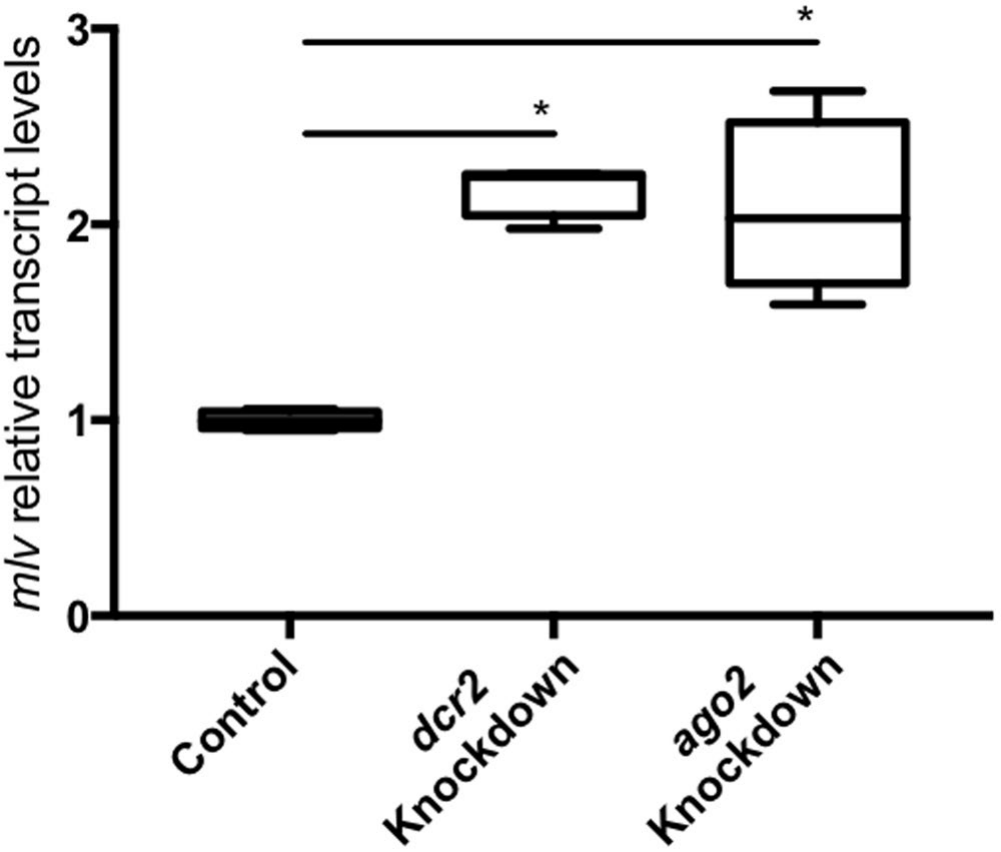
Knockdown of *dcr2* and *ago2* results in increased *mlv* transcript levels in lepidopteran cells. High Five cells were transfected with ds*dcr2* (*dcr2* Knockdown), ds*ago2* (*ago2* Knockdown) or ds*luc* (Control). The graphs depict box-plots of *mlv* relative transcript levels. Statistical analysis (Shapiro-Wilk normality test and Mann–Whitney test) was performed in GraphPad Prism 7 (*P < 0.05; n = 4).

Since we have demonstrated the presence of virus-specific Ago2-bound siRNAs corresponding to the genomes of three different known persistent viruses in BmN cells (Fig. 4), we further investigated the importance of key siRNAi pathway players in the control of *mlv* transcript levels in lepidopteran cells. Therefore, we made use of the High Five cells persistently infected with MLV, investigated under previous sections. We knocked down *dcr2* and *ago2* in these cells via RNAi and assessed the *mlv* transcript levels. The knockdowns were shown to be efficient in both situations (Fig. S6, supplementary data). When *dcr2* or *ago2* were downregulated, significantly increased *mlv* relative transcript levels were observed (Fig. 5).

## Discussion

In the current study, we demonstrate that boosting the expression of certain RNAi components in lepidopteran cells results in the improvement of the defence against an acute viral infection. More specifically, overexpression of the key siRNAi factors Dcr2 and Ago2 in High Five cells results in a strong reduction of CrPV-induced cell mortality (Fig. 1). Importantly, impressive *in vivo* improvements of antiviral capacity against the BmNPV have already been demonstrated in transgenic *Bombyx* strains expressing virus-specific dsRNA. This emphasizes the advantage of using transgenic moths with higher antiviral capacity in sericulture, an industry where lepidopteran viral diseases cause significant losses^31–33,41^. In this context, our work suggests that overexpression of siRNAi components is a potential alternative and/or complementary approach to obtain enhanced antiviral response in Lepidoptera. Although the proposed strategy remains to be tested *in vivo*, it is important to note that overexpression of Ago2 in *B. mori* larvae induces an enhanced RNAi response. This is in line with what has been observed in cell lines and, together with the here reported results, raises the possibility that an improved antiviral response might also be obtained *in vivo* by overexpressing key siRNAi factors^29,30^.

Since overexpression of Dcr2 and Ago2 led to an enhanced antiviral response, we wondered whether upregulation of these key siRNAi components could constitute a natural cellular response upon an acute infection of CrPV. However, although a transcript upregulation of both *dcr2* and *ago2* was observed upon infection, the observed effect was very moderate (Fig. 2). In addition, 24 h.p.i. the transcript levels returned to basal. Therefore, although these data do not allow to clearly conclude that an upregulation of *dcr2* and *ago2* constitutes an active cellular defense strategy in the case of a CrPV acute infection, it is possible to conclude that the RNAi-genes are differentially regulated during the infection. In accordance with these results, differential regulation of RNAi-genes, including *dcr2* and *ago2*, has been reported in different tissues of the bumblebee upon infection with the Israeli Acute Paralysis Virus^42^. In this context, it is relevant to discuss the possible triggers leading to the differential regulation of RNAi genes upon viral infection. Interestingly, delivery of long dsRNA, an important PAMP for viral infections, irrespective of the sequence, reduces viral infection in bees^43,44^. Moreover, long dsRNA injection induces upregulation of *dcr2* and *ago2* in the moth *Manduca sexta*^45^. However, the potential role of other factors in this process must also be considered. In fact, it has been shown that stress factors can help to strengthen the RNAi-based antiviral response in insects^9,46^ and that differences in the detection processes of viral and other exogenous dsRNAs occur in *Drosophila*^47^. Therefore, it is interesting to consider that dsRNA and other signals derived from stress and/or immune responses might be responsible for the differential regulation of *dcr2* and *ago2* in High Five cells upon CrPV infection. However, further research is required to elucidate this question.

The available knowledge on insect antiviral immunity derives mainly from research regarding acute infections. Yet, the ubiquity of persistent viral infections is currently becoming clear and the potential influence of these infections on several (immune) processes must be considered. For instance, it has been proposed that persistent viral infections have an impact on the highly variable RNAi efficiency observed across insect species^13^. In this scope, although it has been demonstrated that RNAi plays a key role during persistent viral infections in *Drosophila* cells^21^, these processes remain unknown in other species. Thus, unveiling the mechanisms underlying the establishment and maintenance of persistent viral infections in insects is of major interest. In the current study, overexpressing Dcr2 and Ago2 does not result in lower transcript levels of the persistent MLV in the investigated High Five cells (Fig. 3). Nevertheless, by showing that silencing Dcr2 and Ago2 results in increased *mlv* transcript levels in High Five cells (Fig. 5), and by identifying BmN-derived Ago2-bound siRNAs mapping to the genome of three different lepidopteran persistent viruses (Fig. 4), our work suggests a role of the siRNAi pathway in controlling the levels of persistent viral infection in lepidopteran cells.

Here it is important to note that acute and persistent viral infections are characterized by distinct levels of replication, which are higher in acute infections and lower in persistent infections. Therefore, it is interesting to hypothesize that high levels of CrPV (acute virus) replication result in saturation of the host RNAi machinery, which is compensated when Dcr2 and Ago2 are overexpressed. In contrast, the typical lower replication levels of persistent infections do not cause RNAi-machinery saturation, making it tempting to assume that the overexpression of key RNAi factors might be redundant. This hypothesis is also in accordance with the observation that efficient knockdowns of *dcr2* and *ago2* induce an increase in *mlv* transcript levels (Fig. 5). As opposed to the overexpression approach, a knockdown of key RNAi-factors appears to be sufficient to disrupt the equilibrium between the RNAi-based antiviral machinery of the cell and the viral transcript levels. This result strongly indicates that both Dcr2 and Ago2, key RNAi players, are crucial to maintain stable, low *mlv* transcript levels in High Five cells. Still in the context of these observations, it is relevant to note that CrPV encodes the well-described RNAi inhibitor 1 A. Nevertheless, it is still unknown whether MLV encodes a VSR and this possibility cannot be excluded^5,22^. Therefore, it is as yet not possible to evaluate the antiviral efficacy of overexpressing Dcr2 or Ago2 in function of the potential presence of VSRs. In this regard, additional research concerning the existence of a possible MLV-VSR and its potential mode of action will be required.

To conclude, in this study we have employed a novel acute infection model consisting in the infection of *T. ni* High Five cells with the well-described CrPV; and of the previously reported persistent viral (MLV) infection system in High Five cells. We have demonstrated that overexpression of Dcr2 and Ago2 is sufficient to reduce the CrPV-induced mortality in High Five cells. In addition, by using an integrated approach in BmN and High Five cells, we propose a role of the siRNAi pathway in controlling the level of persistent viral infection in these lepidopteran cells.

## Material and Methods

### Sequence retrieval, protein domain prediction and phylogenetic analysis

By using the Dcr2 and Ago2 sequences of other insects as query, transcript sequence information for *T. ni dcr2 and ago2* was retrieved from NCBI, accession number SRA057390^39^, with tBLASTn. The deduced amino acid sequences were determined using ExPASy translate tool, which were then used for the prediction of protein domains using NCBI Conserved Protein Domain Search.

Next, specific protein conserved domains, namely PAZ for Dicer proteins and PIWI for Argonaute proteins, were aligned using Muscle alignment (MEGA7) and used for the construction of a maximum likelihood (ML) phylogenetic tree with 100 bootstraps (MEGA7). The PAZ domain of a Dicer sequence of a phylogenetically distant organism, namely *Arabidopsis thaliana*, was used as outgroup. In the case of Argonautes, the PIWI domain of *Schizosaccharomyces pombe* was used as outgroup.

### Cell culture and transfections

The Bm5 and High Five cell lines were maintained in a complete medium consisting of IPL-41 Insect Medium (Sigma-Aldrich), supplemented with 10% heat-inactivated fetal bovine serum (Sigma-Aldrich), 0.25 μg/ml of amphotericin B (Sigma-Aldrich), 100 U/ml penicillin and 100 μg/ml streptomycin (Gibco, Life Technologies). The cells were subcultured weekly and maintained at 27.5 °C.

Bm5 and High Five cells were transfected using Escort IV (Sigma-Aldrich), according to manufacturers’ instructions. An optimized volume of the transfection reagent was used, namely 3.7 μl and 15 μl per well of a 24- and 6-well plate, respectively.

In the overexpression experiments, the expression constructs were transfected overnight, at a concentration of 1 μg/ml. In addition, the pBmIE1 helper plasmid encoding the *ie-1* gene for *B. mori* nuclear polyhedrosis virus^48^ was used, at a concentration of 0.3 μg/ml. In the knockdown experiments, the dsRNA was transfected at a concentration of 2.5 μg/ml. After the transfections, the medium was replaced for the complete medium above mentioned.

### Expression constructs

The constructs for overexpression, namely pEA-Myc-BmDcr-2 and pEA-BmAgo-2-MycHis, as well as pEA-pac, that contains the ORF (open reading frame) of puromycin resistance gene (negative control), were previously described by Kolliopoulou and Swevers^29^.

### Protein extract preparation and Western blotting

Transfected Bm5 and High Five cells were collected and lysed by sonication (Branson) in a lysis buffer consisting of Tris-Cl 50 mM, NaCl 150 mM, EDTA 1 mM, Triton-X-100 1%, Sodium Dodecyl Sulphate (SDS) 0.5% and Protease Inhibitor Cocktail Tablets Complete (Roche). Next, the total amount of proteins was quantified by means of a Bicinchoninic acid assay, after which 18 μg of each sample were separated by SDS-polyacrylamide gel electrophoresis. Then, the proteins were transferred to a Trans-Blot Turbo Mini PVDF membrane using the Trans-Blot Turb Blotting System (Bio-Rad). The blots were washed and blocked with a skimmed powder milk solution 5% for 2 hours. Anti-c-Myc antibodies produced in rabbit (Sigma-Aldrich) were diluted (1:5000) and incubated with the blots overnight at room temperature. Washing was then performed, followed by 2 hours of incubation with Polyclonal Goat Anti-Rabbit Immunoglobulins/HRP (Dako), diluted 1:50000. Finally, the blots were washed and the detection was performed with Super Signal West Dura Extended Duration Subtract Kit (Thermo Scientific). The chemiluminescent bands were visualized using a ChemiDoc™ MP Imaging System with Image Lab Software (Bio-Rad).

### Synthesis of dsRNA

Double stranded RNA constructs for *Tn-dcr2* (480 bp) and *Tn-ago2* (497 bp) were synthesized using the MEGAscript RNAi kit (Ambion). A DNA template flanked by two T7 promoter sequences was synthesized. Therefore, a PCR reaction was performed with cDNA of High Five cells, gene-specific primers containing a T7 promoter sequence at the 5′ end (Table S1, supplementary data), and REDTaq mix (Sigma-Aldrich) as a source of DNA Taq polymerase, dNTPs and PCR buffer. The amplification products were subsequently analyzed by 1% agarose gel electrophoresis and then visualized under UV-light with the ProXima 2500 (Isogen Life Science). Moreover, the template sequences were validated by first cloning the fragments into the pCR4-TOPO vector by means of the TOPO TA Cloning Kit for Sequencing (Life technologies) and subsequently sequencing the inserted DNA fragments by Sanger Sequencing (LGC, Berlin, Germany). The PCR product was used directly as template for production of dsRNA.

Synthesis of *luciferase* dsRNA was performed using a pLitmus 28i vector (New England Biolabs) containing a 513 bp fragment from the ORF of firefly *luciferase*^49^. This vector was linearized by EcoRI or HindIII and subjected to RNA synthesis.

The MEGAscript RNAi kit (Ambion) was used for the synthesis and further purification of the dsRNA, according to manufacturers’ instructions. Both *luciferase* RNA strands were first synthesized independently before being mixed to anneal, while transcripts made from a single template with opposing T7 promoters (for ds*dcr2* and ds*ago2*) were hybridized during the transcription reaction. The final dsRNA integrity and concentration were assessed by gel electrophoresis using a 1% agarose gel and by means of a Nanodrop spectrophotometer (Thermo Fisher Scientific).

### Production and Quantification of Cricket Paralysis Virus

The CrPV suspension was produced in cultured *D. melanogaster* S2 cells as previously described^50^ and purified by ultracentrifugation in a sucrose cushion. The final viral pellet was resuspended in phosphate-buffered saline (PBS). The viral concentration was determined by Transmission Electron Microscopy, negative staining, by CODA-CERVA (Ukkel, Belgium).

### RNA extraction

High Five cells were harvested by means of a cell scraper, pelleted and snap frozen. The RNA was extracted with the miRNeasy Mini Kit (Qiagen), as described in the correspondent protocol. A DNase treatment (RNase-free DNase set, Qiagen) was performed to eliminate potential genomic DNA contamination. Quality and concentration of the extracted RNA were assessed using a Nanodrop spectrophotometer (Thermo Fisher Scientific).

### Quantitative real time PCR (qRT-PCR)

The cDNA synthesis was performed using the PrimeScript First Strand cDNA Synthesis Kit (TaKaRa) following manufacturer’s specifications, with equal amount of total RNA for every sample. Upon synthesis, the cDNA was diluted 25.5 times with MilliQ water.

The qRT-PCR primers are displayed in Table S2 (supplementary data). The efficiency of each primer pair was assessed by designing relative standard curves for gene transcripts with a serial dilution (5x) of cDNA. In addition, a dissociation protocol was performed to detect the presence of primer dimers as well as amplification of a single PCR-product. Each PCR reaction was performed in duplicate and contained 5 μl of SYBR Green solution (Invitrogen), 0.375 μl of 10 μM forward primer, 0.375 μl of 10 μM reverse primer and 4.25 μl of cDNA.

For each experiment, the stability of candidate housekeeping genes was assessed with the geNorm program^51^. In the experiment represented in Fig. 2, *tubulin* and *ef1a* were selected; in the experiment depicted in Fig. 3, *rps18* and *ef1a* were used; in the experiments represented in Figs 5 and S6, *elf4a* and *ef1a* were chosen. The transcript levels of the selected reference genes were used to normalize the measured transcripts of interest. In every experiment a no-template control was included. The PCR reaction was performed and analyzed in a 96 well plate using the StepOne System (ABI Prism, Applied Biosystems). The relative transcript quantity was calculated according to the delta–delta Ct method.

### CrPV-cell infection and viability assays

High Five or Bm5 cells were collected by centrifugation (8 minutes at 1000 g), resuspended in the CrPV suspension, or PBS (control), diluted in IPL-41 Insect Medium (Sigma-Aldrich) and incubated at room temperature during 2 hours in a shaker plate. Then, following a washing step in IPL-41 Insect Medium (Sigma-Aldrich), the cells were resuspended in complete medium, plated in 24-well plates, and maintained at 27.5 °C. At the selected time points, the cells were harvested by means of a scraper and collected for further analysis. The assessment of viability was performed using a trypan blue assay. Therefore, equal volumes of 0.4% trypan blue solution (Sigma-Aldrich) and cell suspension were mixed and loaded on a counting chamber. The determination of the number of living and dead cells was performed manually at an inverted microscope (Leitz Wetzlar).

### Detection of MLV

MLV was detected via PCR using cDNA from High Five cells as template. The reaction was performed with REDTaq ReadyMix PCR Reaction Mix (Sigma Aldrich), using *mlv*-specific primers (Table S3, supplementary data) and the product was analyzed on a 1% agarose gel electrophoresis. A 100 bp ladder was used (GeneRuler, Thermo Scientific).

### Analysis of viral-derived siRNAs bound to Ago2 of BmN cells

A publically available library of Ago2-bound small RNAs (SRX201604, GMS1025527_Unannotated_smal_RNAs) was downloaded from NCBI^40^. This library was mapped to the genome of *B. mori* (ASM15162v1, GCA_000151625.1) and only the unmapped reads were selected for further analysis. The mapping was performed with Bowtie2, using the sensitive preset parameters, as described in the corresponding manual. In order to identify persistently infecting viruses, the library was analyzed based on previously reported methodology^17^. Shortly, in order to assemble the small RNAs into contigs, the Velvet program was used^52^, with a K-mer value of 17. The obtained contigs were used to perform a BLASTn search against all publicly available viral genome sequences (downloaded from NCBI) using the Blast2GO program. In order to directly map the small RNAs reads into the viral genomic sequences, Bowtie2 was used as described before. The graphs depicting the size distribution of the mapped reads, as well as the genomic distribution of the siRNAs reads (18–24 bp) were obtained in R with the viRome package.

### Data Availability Statement

The datasets used and analyzed during the current study are available in the NCBI repository:

- accession number SRA057390 http://www.ncbi.nlm.nih.gov/sra/?term SRA057390
- accession number GMS1025527 https://www.ncbi.nlm.nih.gov/geo/query/acc.cgi?acc=GSM1025527
- accession number GCA_000151625.1 https://www.ncbi.nlm.nih.gov/assembly/GCF_000151625.1/.

## Electronic supplementary material

Supplementary material
