# Supplementary material for: Insights into RNAi-based antiviral immunity in Lepidoptera: acute and persistent infections in Bombyx mori and Trichoplusia ni cell lines
Source: Sci Rep. 2018 Feb 5;8:2423. doi: 10.1038/s41598-018-20848-6 (PMC5799340; doi:10.1038/s41598-018-20848-6)
Supplement: Supplementary file 1 — Supplementary material [file 41598_2018_20848_MOESM1_ESM.pdf]

Insights into RNAi-based antiviral immunity in Lepidoptera: acute and persistent infections in *Bombyx mori* and *Trichoplusia ni* cell lines

Dulce Santos[$1*], Niels Wynant[$1], Stijn Van den Brande[1], Thomas-Wolf Verdonckt[1], Lina Mingels[1], Paulien Peeters[1], Anna Kolliopoulou[2], Luc Swevers[2], Jozef Vanden Broeck[1]

[$]These authors contributed equally to this work

[*]Corresponding author: Dulce Santos, dulce.cordeirodossantos@kuleuven.be

Affiliations:

[1]Molecular Developmental Physiology and Signal Transduction Research Group, Animal Physiology and Neurobiology Division, Department of Biology, KU Leuven, Naamsestraat 59, box 2465, 3000 Leuven, Belgium

[2]Insect Molecular Genetics and Biotechnology, Institute of Biosciences and Applications, National Center for Scientific Research "Demokritos", 153 10 Aghia Paraskevi Attikis, Athens, Greece

Supplementary material

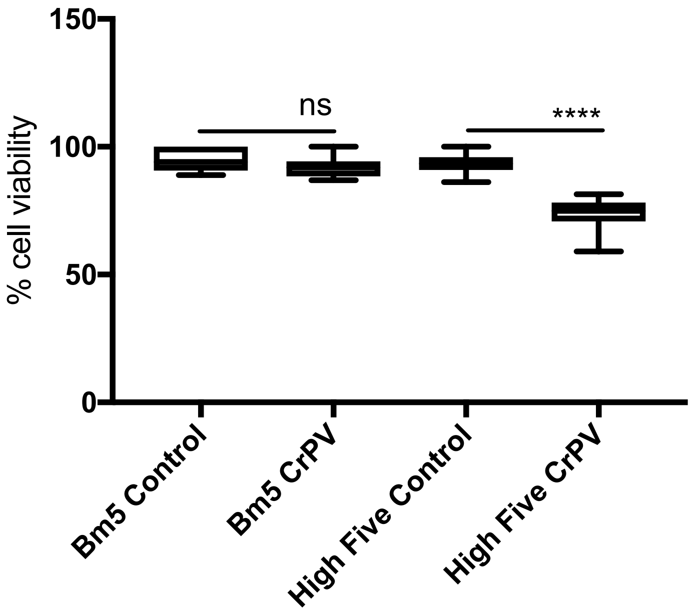

Figure S1: High Five cells are more sensitive to CrPV infection than Bm5 cells. Bm5 and High Five cells were infected with CrPV, MOI25, or treated with PBS (Control). Mortality was assessed after 24h. The graph depicts box-plots of the cell viability of each group, in percentage. Statistical analysis (Shapiro-Wilk normality test and Unpaired T-tests) was performed in GraphPad Prism 7 (**** $P<0.0001$; $n=12$).

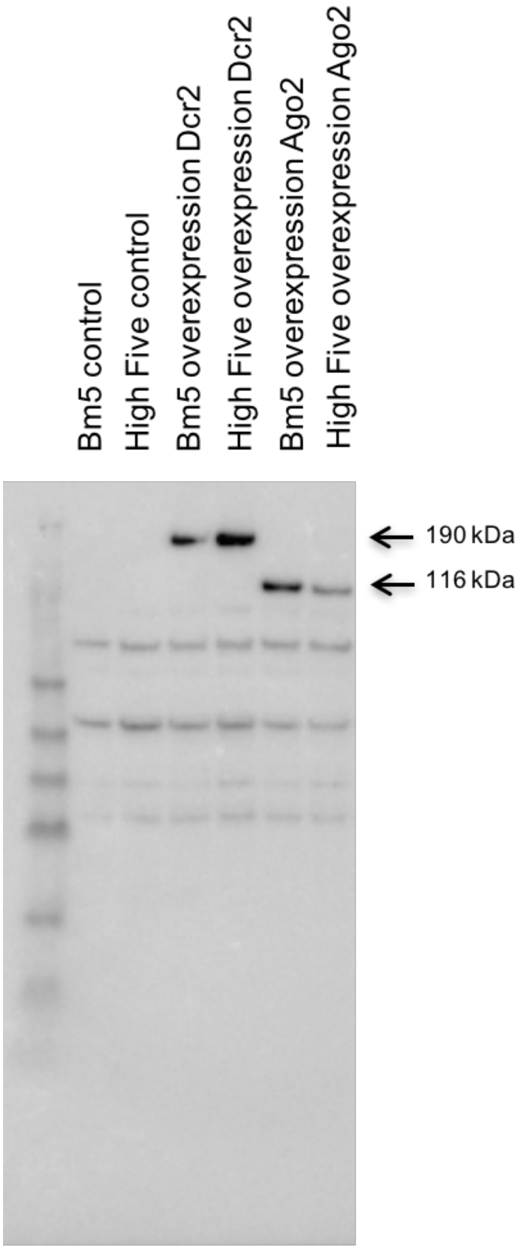

Figure S2: Overexpression of *B. mori* RNAi factors in Bm5 and High Five cells. A western blot analysis performed with an antibody specific to the Myc tag of the overexpressed proteins confirmed the overexpression of *Bm*-Dcr2 (190 kDa) and *Bm*-Ago2 (116 kDa) in Bm5 and High Five cells transfected with with the pEA-pac control vector. Lane 1: SeeBlue Plus2 Pre-Stained Protein Standard ladder (Novex).

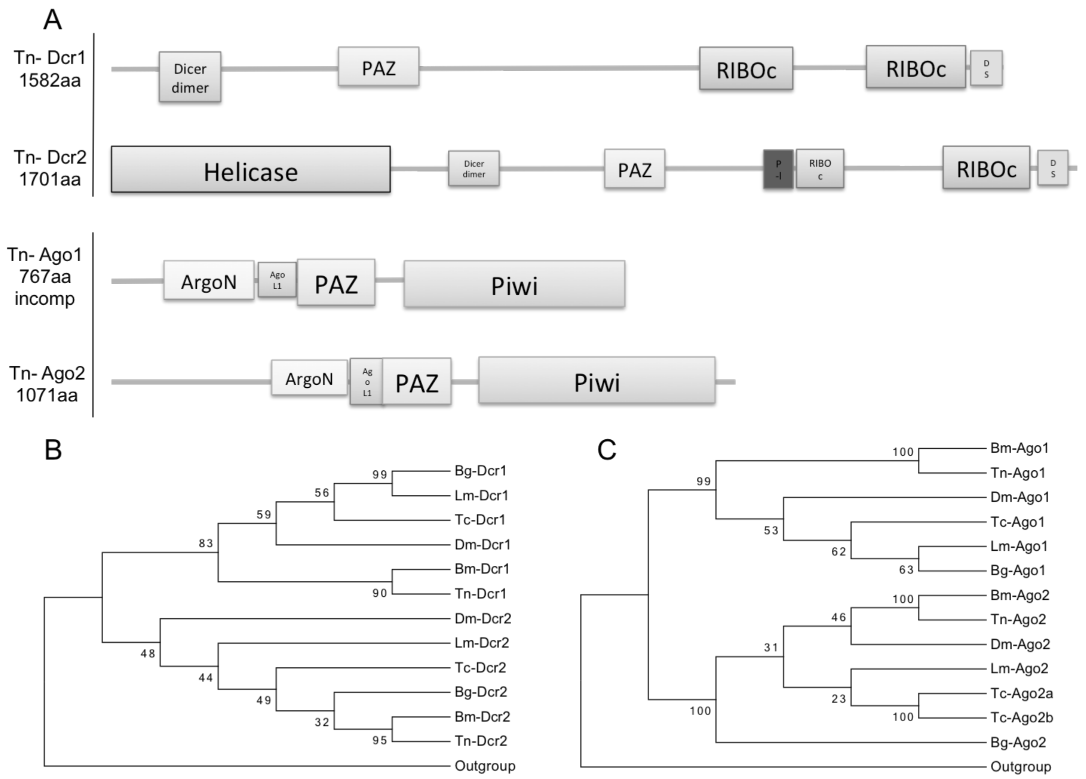

Figure S3: Identification of *T. ni* Dicer and Argonaute proteins. A: Protein domain prediction of *Tn*-Dcr1, *Tn*-Dcr2, *Tn*-Ago1 and *Tn*-Ago2. B: Maximum likelihood phylogenetic tree (100 bootstraps) with the amino acid sequence of the PAZ domain of Dicer proteins of *B. mori* (*Bm*-Dcr1 and *Bm*-Dcr2), *Blattella germanica* (*Bg*-Dcr1 and *Bg*-Dcr2), *D. melanogaster* (*Dm*-Dcr1 and *Dm*-Dcr2), *Locusta migratoria* (*Lm*-Dcr1 and *Lm*-Dcr2), *Tribolium castaneum* (*Tc*-Dcr1 and *Tc*-Dcr2), and *T. ni* (*Tn*-Dcr1 and *Tn*-Dcr2). For the outgroup, the PAZ domain a Dicer sequence of a phylogenetically distant organism, namely *Arabidopsis thaliana*, was used. C: Maximum likelihood phylogenetic tree (100 bootstraps) with the amino acid sequence of the PIWI domain of Argonaute proteins of *B. mori* (*Bm*-Ago1 and *Bm*-Ago2), *B. germanica* (*Bg*-Ago1 and *Bg*-Ago2), *D. melanogaster* (*Dm*-Ago1 and *Dm*-Ago2), *L. migratoria* (*Lm*-Ago1 and *Lm*-Ago2), *T. castaneum* (*Tc*-Ago1, *Tc*-Ago2A and *Tc*-Ago2B), and *T. ni* (*Tn*-Ago1 and *Tn*-Ago2). For the outgroup, the PIWI domain an Argonaute sequence of a phylogenetically distant organism, namely *Schizosaccharomyces pombe*, was used. (Helicase: helicase insert domain; Dicer dimer: dicer dimerization domain; PAZ: PAZ domain; RIBOc: Ribonuclease III C terminal domain; DS: Double-stranded RNA binding motif; ArgoN: N-terminal domain of Argonaute; P-l: P-loop NTPase superfamily domain; incomp: incomplete sequence).

A

```
Bm-Dcr2         MTTTEGI-QYHEQKARPYQAILEEIASSKNTIIHLPTGSGKTFIAQRLIKKFRNQLKKPW
Tn-Dcr2         MDVDDGSDGVEQLKSRPYQAQLEEIAVRNNTIIYLPTGSGKTFIAICLIKRFREALQLPW
                * . :*    .: *:***** *****  :****:***********  ***:**: *: **

Bm-Dcr2         GEGGKRSFFLVNTVPLVNQQKNVIEMMCPVDGVGAYSSEDGVDYWKKADWDSELARNQVI
Tn-Dcr2         GQGGKRTFFLVNNVPLVTQQKNVIEKLCAVSGVGAYSSENGVDYWDKAKWDAELEKNQVI
                *:****:*****.****.******* :* *.********:*****.**.**:** :****

Bm-Dcr2         VMTSQILSDMLTHAYIRIEDINLIIFDECHHAVEDHPMRVIMKHFLNCKAHEQPRVLGLT
Tn-Dcr2         VMTSQILNDMLTHQYIRIEDINLLIFDECHHAVEDHPMRVIMKHFEGCPKHSQPRVLGLT
                *******.***** *********:********************* .*  *.********

Bm-Dcr2         ATLLNGNVSISKIEETLKQLENTFHATIATVDDLGEVMTYSTNPQEFIQYFRKPQMSEAA
Tn-Dcr2         ATLLNANVKSRKVEDTLHDLEITFHATIATVDELGKVLNYSTNPNEMVQFYRSSPPSAVT
                *****.**.  *:*:**::** **********:**:*:.*****:*::*::*.   * .:

Bm-Dcr2         KEAISLLNTLQNLVMSVKLPVSLPSKDFKKTPCQRDISNDPNKIVKAVKNLIVGMITNLE
Tn-Dcr2         NEVIKLLSVRQELIASVKLPRSSTKQTITLKQHQEDISNNPKKIVKAVKNMISSMILFLN
                :*.*.**.. *:*: ***** *  .: :. .  *.****:*:********:* .**  *:

Bm-Dcr2         EMGLYGGSLGILAYIVLLERLKRKASTKEENLLYSTVITHSTEARMILLDAMENETGYDK
Tn-Dcr2         ELGMYGGSLGILAYIILLERLRRRASSKEEDILYQNVITCCIDARARLLKAMSDVHGYER
                *:*:***********:*****:*:**:***::**..*** . :**  **.**.:  **::

Bm-Dcr2         IIKHSSEKILQLIDLLKEYNPLILEKPGELLKVNHSRKPLCGIIFTKQRFTSKILYNILM
Tn-Dcr2         IIKYSSEKVLLTLNILKEYNPAYQDTPGVLLKVNRSRKPLSAIIFTKQRFTAKVLYNLLK
                ***:****:*  :::******   :.** *****:*****..*********:*:***:*

Bm-Dcr2         DLKKARPDEFEFLKHDFVVGFSINPFKSTREEHYLKKSSQKAILGFNNGDLNCLISTSVI
Tn-Dcr2         DVRDSNPAEFDFLKHDFVVGFNVNPLKSTREEYYIKKTGQQALLKFGNNDLNCLISTSVI
                *::.:.* **:**********.:**:******:*:**:.*:*:* *.*.***********

Bm-Dcr2         EEGLDIPQCALVVRYDAPTEYRSYIQSKGRARSSE---------ASFVILVDIEDKQSFL
Tn-Dcr2         EEGIDIPQCLLVLRYDQPLEYRSYIQSKDRYRTHGLWCKCNRLEQTRPLTINREDEKKFM
                ***:***** **:*** * *********.* *:            :  : :: **::.*:

Bm-Dcr2         IKYDAFQKTEERIRNLIVGD-DSRAAPTTQDIHENLYDDDEVPPYETSNGNILYSVSAIS
Tn-Dcr2         TLYKEFQETEQLIQRILVGNTDDRDEPAQENIDKNLYQDEDVPPFISPYGGRLTATSAIS
                  *. **:**: *:.::**: *.*  *: ::*.:***:*::***: :  *. * :.****

Bm-Dcr2         LLSRYCSILPHDQFTTITPVWLQEVVENGAK---KQVTVILPIGSPVKEEIKGDPYSNTK
Tn-Dcr2         LLNRYCSMLPHDHFTIITPMWIKETVTNKHGFDCNLVTIVLPIACPIKEEIKGMPMYNLK
                **.****:****:** ***:*::*.* *      : **::***..*:****** *  * *

Bm-Dcr2         TAKRSAALKACIKLHELGELHPLTLLPYKYAEVDYERDDIRGCFPNWTKDCETEI-KENV
Tn-Dcr2         SAKRSAALNACVKLYEAGELDPLTMLPMRYTAVDFDDADVQSCFLNWRNDDMKRVDDPDY
                :*******:**:**:* ***.***:** :*: **::  *::.** ** :*  ..: . :

Bm-Dcr2         PKPGSKKRKRKHKIQFPSHLDAVPDSSVFYLHVIKMEAAFEKPNDNREKALYDFIKRNEC
Tn-Dcr2         PAPGTKGRVRKHRIQFPAVLDSVPDESEYYLHIIKTTTAFAEPKDTREKALYDLLHRPEG
                * **:* * ***:****: **:***.* :***:**  :** :*:*.*******:::* *

Bm-Dcr2         YGFLTQKPLPKLCEFPAFTSVGEITTSIEVNYAVIKLTPIIFELVKQFHYFLFDQILAIN

Bm-Dcr2         LWYKATLLPSIIHRVHMLLVAHELLVEISNATKYGQPILKKTESFQPIQVDRYIAINSLL
Tn-Dcr2         LWYKAIMLPSIVHRVFMLLVAHELLTEISESTKYGNPKRRKGEEWRPVSSNMQIATLSLL
                ***** :****:***.*********.***::****:*  :* *.::*:. :  **  ***

Bm-Dcr2         SQVEEPLHVHTIDRIHKGTDDCL---KKVVPLKKSLYKLQRKKIDKEYPWDQRIEPIDVE
Tn-Dcr2         AQVEEPTPITSVDRINNPTDDENPRRPNIMSIKQSLYQLQQKKLSKDYPWDEKMEPIDIE
                :*****  : ::***:: ***      ::: :*:***:**:**:.*:****::****:*

Bm-Dcr2         RNLSTITLMDIECYDEFVSAPISSASN--NPCSPPRIVPK---APAIAPPPIKYVDKIEI
Tn-Dcr2         RNLSTVTVMDIECYDEFVSSPLVPIMSPTRVLSPPRVVVGSKISAAISAPPAKYNDKLNI
                *****:*:***********:*:    .  .  ****:*     : **: ** ** **::*

Bm-Dcr2         LKKTPTGRGPELRDVLAALTTINSNDTFDLERPETLGDSFLKFAASLYLHHKFPNMNEGQ
Tn-Dcr2         LKMTATGNGPELRDILTALTTIKSHDTFNLERVETLGDAFLKFAASLYLFHKFPKFNEGQ
                ** * **.******:*:*****:*:***:*** *****:**********.****::****

Bm-Dcr2         LTNIKGKLISNRNLYYAGAKFNLGGRMKVEQFSPRSDFLVPGFFAPPKLEEFIEQRQIRP
Tn-Dcr2         LTNIKGRLISNRNLYYAGERFNLAGRMKVEQFSPRKDFMVPGYFAPPEVEKFIAEKKLRP
                ******:*********** :***.***********.**:***:****::*:** ::::**

Bm-Dcr2         TFLIGIQFTEEEVMTGELSEESWESVMNNFQ--NGIAEAEPEGCAQNSMQCYVHSQAVAD
Tn-Dcr2         TFLIGVYFPSSEAFDGNLSKESMAMVRDRFADCDGTAETEPECRVQNAMQLYIHSQAVAD
                *****: * ..*.: *:**:**   * :.*   :* **:***  .**:** *:*******

Bm-Dcr2         KSIADSVEALIGTYLLSGGILAAVKLLEWMEVFPPQDNFADMLHKPVQTPLSKNLATEAD
Tn-Dcr2         KSVADCVEALIGTYLLSGGVLGAIKVIEWMRIIPPQDNFATYLHTRVSTVLSEKRATESD
                **:**.*************:*.*:*::***.::*******  **. *.* **:: ***:*

Bm-Dcr2         IDFLLNNSRTDVEKILNYTFKDSTFLLNALSHSSYIRNRLTSSYERLEFLGDAIIDFLVT
Tn-Dcr2         INFLLSHCRPDVEKILNYKFKDPSHLLEALSHPSYIRNRLTRSYERYEFLGDAILDFLIT
                *:***.:.* ********.*** :.**:**** ******** **** *******:***:*

Bm-Dcr2         SHIFENCRELKPGEMTDLRSALVNNVTFAAYTVKLGLHKYLCSELNPSLEKAIMKFVEHQ
Tn-Dcr2         SHVFENCGDLKPGEMTDLRSALVNNVTFASYVVKLGLHKFLCSELNPTLDKAVITFVDHQ
                **:**** :*******************:*.*******:*******:*:**::.**:**

Bm-Dcr2         IERNHEIEFDVLLLISEVDCQVAEYVDVPKVLSDIFEALVGAVYLDSGGNLETVWSVLYK
Tn-Dcr2         VQREHQIVEDVLYLIDEEECHIAEYVEVPKVLSDIFEALVGAIFLDSGGDLQTVWALVYR
                ::*:*:*  *** **.* :*::****:***************::*****:*:***:::*:

Bm-Dcr2         IMHVEIDSFSKRIPKQPVKILYEMIHACPVFEKQIVVNPDIPKVMVPVSFTKNDQRLAAY
Tn-Dcr2         IMCKEIHAFSSRIPQQPVKVLYEKIHACPVFDKSVVIDPDIPKIKVGVTITKNDWQHTVY
                **  **.:**.***:****:*** *******:*.:*::*****: * *::**** : :.*

Bm-Dcr2         GVGVNKSQAKRAAAKLALKILDS
Tn-Dcr2         GVGKNKSQAKRAAAKMALKVLGI
                *** ***********:***:*.
```

```
B
Bm-Ago2         -MARGKNKGGKKEAPDSTKTPSSESQPSESQPSTSQIP----------------------
Tn-Ago2         MGKQGKKKGGKKEPQKEESTSSHVS----QQPSTSAQDEEEEEVGLTGAARKRKEKKELK
                   :**:******  .. .* *  *    .*****

Bm-Ago2         ----------------------------------------------------TTEPTTTI
Tn-Ago2         EKAKQTLQALQSTRKTTTSVSEVPSEESSRAGSEAPSEAKPEPGPSKQATPPPDLKVEEI
                                                                        .  *

Bm-Ago2         EDDLGGLGLGESRKRRPRKKPTEKQESLAQAELSNPKLTQTDNPKAEVPKTEAPKTEALK
Tn-Ago2         IEEFEGLGLPSTKKKKPKKKKPGEVTSPTEAPKAA--------P-AQVPPT---------
                 ::: ****  .::*::*:**   :  * ::*  :         * *:** *

Bm-Ago2         PEAPIPEACKSEAPKSEESKIETRGSKPEAAADKPQDDDDGLGLGLG-GGGRKKTRSRKP
Tn-Ago2         -------AAA------QESSAA-VVSCPPWSGPEPADLSSPLSWGPPPGKGRPRGRPVQL
                       *.       :**.     * *  :. :* * .. *. *   * ** : *  :

Bm-Ago2         KFTAVETDIKYSKAPSSEPAIPGPSQS-KPITSTASQPIQYVQNKPEVKAAPAAPVLYKI
Tn-Ago2         PSPQIPTTRTFPLSPSQSGASSVPSSALSPTSS--SVSVSGSERRPPR-SLEPVLCRYKI
                    : *  .:  :**.. *   **.: .* :*  *  :.  :.:*   :   .   ***

Bm-Ago2         PDKILSP--PSRTVPILTNYLAMKITKPLKIYRYDVTFKPDKPKKFIAQVFKLVKSKEFP
Tn-Ago2         PMKIPTRTVRARNITVLANYLEMSF-KSIEISRYDINFSPDRPKKMLPIVFQQVKKRFYA
                * ** :    :*.: :*:*** *.: * ::* ***:.*.**:***::  **: **.: :

Bm-Ago2         KEILAFDQTKNCYSLTPLPKIT-TERYGVKVVIKDMNGKDMPFEVSFKASGIVDYNNVLK
Tn-Ago2         SDLIAFDQMKNCYSLRPLKNVTATERFTTTVDLLDQNGRNMTFEVTIKSTGVVDLGNIKR
                .:::**** ****** ** ::* ***: ..* : * **::* ***::*::*:** .*: :

Bm-Ago2         HMATGGSSLNAPTDTIQCIDIVLKQGTLESYVKAGRQYFMRPASPIDLGDGLEMWTGLFQ
Tn-Ago2         YMVERGSSLCHPTEEIQCIDVILRQGALESYVKAGRQFFKRPANPVDLGSGYEMWTGLFQ
                :*.  ****  **: *****::*:**:**********:* ***.*:***.* ********

Bm-Ago2         SAIFTSKAFINVDVAHKGFPKNQPMIDAFTRDFRLDPNRPVDRQPGRAAEAFNEFIRGLK
Tn-Ago2         SAIFTNKSFINIDVAHKGFPKAQSMIDCLVKDFNLDPYRPIDNQRG--GDNFATFVKGLK
                *****.*:***:********* * ***.:.:**.*** **:*.* *  .: *  *::***

Bm-Ago2         VVSKILGTGPSSGQLREHICNGVVDPPSRQTFTLENDKGPPVRMTVYEYFMKEKKYRIKY
Tn-Ago2         VVASLVGNTATAGHKREFVCNGVVGPPDKLTFPITESDGRSRKLTVAEYFAKEKQYRLKY
                **:.::*.  ::*: **.:*****.**.: ** : :..*   ::** *** ***:**:**

Bm-Ago2         PDLNCLWVGPKDKNIYLPMELVEVAYGQARNKQLNDRQLSTMVREAATPPDVRKRKIEEV
Tn-Ago2         PHLNCLWVGSRERCIYFPMELLNVSYGQPLARQLNEMQVSKMVKEAATPPDERLAKIKEV
                *.******* ::: **:****::*:***   :***: *:*.**:******* *  **:**

Bm-Ago2         IQKMNYSKNQFFKTYGLEIANEFYQVEAKILEAPTLEVGPRQFTVPKKGVWQANCLLKPE
Tn-Ago2         ISNMQYSQNKDFKQFGLEISDKFYTVQAKILDPPVLEVGNAKV-TPRKGQWQANRLLKAE
                *.:*:**:*: ** :****:::** *:****: *.****  :. .*:** **** *** *

Bm-Ago2         ALNSWGFIAIELDPRGCNYEDIVSKLMNTGRQMGMNVTQPKMACFNIRINDLHKSMLHAL
Tn-Ago2         ALQSWGLIAVDTDPR-IDYDSMISLIISTGNQLGMNVSKPKFVNLGARMTSLHGILMNA-
                **:***:**:: ***  :*:.::* ::.**.*:****::**:. :. *:..**  :::*

Bm-Ago2         EKQVNFLVVVVSGRGRDYYHKLKQIAELKVGILTHVFKEDTATRRMNPQTARNILLKVNS
Tn-Ago2         YKDVRFVFVIVSARGRDDYHKVKQMAEREVGILTQCIREMT-ARRINQMTAKNILLKVNS
                 *:*.*:.*:**.**** ***:**:** :*****: ::* * :**:*  **:********

Bm-Ago2         KLMGINQALENRSIPQCLKGGAVMIVGADVTHPSPDQSNIPSIAAVTASMDTKCYIYNIE
Tn-Ago2         KLMGVNQAIEHNTLPKCLRDGNVMIVGADVTHPSPDQSNVPSIAAVTASIDPRCYMYNIE
                ****:***:*:.::*:**:.* *****************:*********:* :**:****

Bm-Ago2         LSIQTPKKEMIVQFEDIMVDHFHAFKKSQGILPKKVFVFRDGVSEGQFAEVMKSELTGLH
Tn-Ago2         LSVQTPKKEMIVEFEDMMFDHLKVYKDRNNSLPKKIFVFRDGVSEGQFAQVMNSELQAVQ

                **:*********:***:*.**::.:*. :. ****:*************:**:*** .::

Bm-Ago2         RAYQRVAGLNAKPEVLFILVQKRHHTRFFLPGNNARFNVDPGTVVDRDIVHPRELDFYLV
Tn-Ago2         QAYQRMAGQSRKPEILFLLVQKRHHTRLFCDGT-SRYNVEPGTVVDTDIVHASELDFYLV
                :****:** . ***:**:*********:*  *. :*:**:****** ****  *******

Bm-Ago2         SHQAIKGTARPTRYHAVCNDGRIPENEVEHLAYYLCHLYARCMRAVSYPAPTYYAHLACL
Tn-Ago2         SHQAIKGTARPTRYHAVCNDGKIPDDEVEQLTYYLCHLYSRCMRAVSYPTPTYYAHLACL
                *********************:**::***:*:*******:*********:**********

Bm-Ago2         RARSLTYGEIFNNNDLEKNPKRLRVLDSMLKQSRMFFV
Tn-Ago2         RARSLTHGERFDNKELERHPKRLHVLDKMLQYSRMFFV
                ******:** *:*::**::****:***.**: ******
```

<u>Figure S4</u>: Alignment of *Bm*-Dcr2 and *Tn*-Dcr2 (A), and of *Bm*-Ago2 and *Tn*-Ago2 (B) amino acid sequences. The alignment was performed with Clustal Omega.

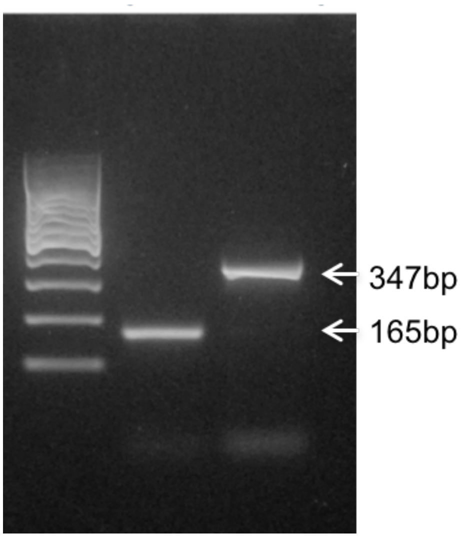

Figure S5: *Bm*-MLV is persistently present in the available stock of High Five cells. The figure depicts an agarose gel electrophoresis of two *mlv*-specific fragments, amplified from High Five cells cDNA. Lane 1: 100bp ladder. Lane 2: fragment of *Bm-mlv* amplified with the primers *mlv*-Fw1/Rv1 (165bp). Lane 3: fragment of *Bm-mlv* amplified with the primers *mlv*-Fw2/Rv2 (347bp).

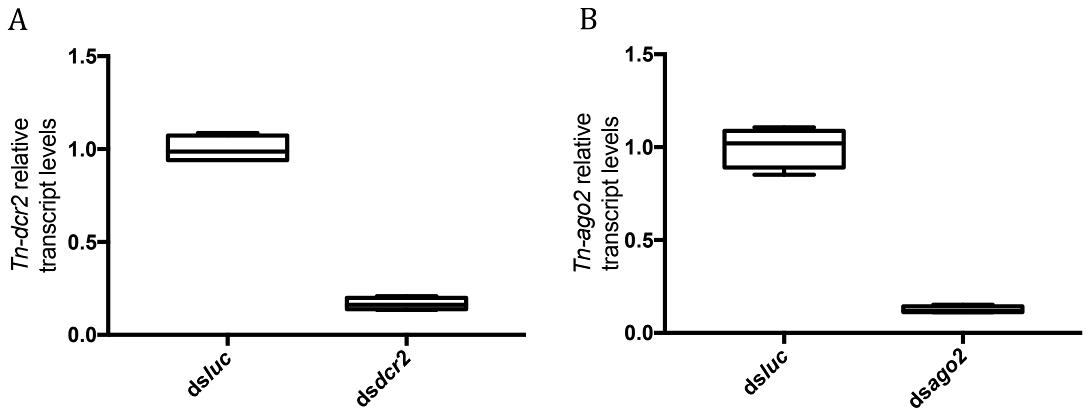

Figure S6: Efficient transcript knockdowns of *dcr2* and *ago2* in High Five cells. Cells were transfected with ds*luc* (control), ds*dcr2* and ds*ago2*. The relative transcript levels of *Tn-dcr2* (A) and *Tn-ago2* (B) were measured after 3 days. The analysis was performed in GraphPad Prism 7 (n=4).

Table S1: Primer sequences used for the production of *Tn-dcr2* and *Tn-ago2* dsRNA. The T7 promoter sequence is shown in bold.

*Tn-dcr2*_T7_Fw	**TAATACGACTCACTATAGGG**GCAGACCAGACGTCGAGAAA
*Tn-dcr2*_T7_Rv	**TAATACGACTCACTATAGGG**CGCTGTCCAGGAATATCGCT
*Tn-ago2*_T7_Fw	**TAATACGACTCACTATAGGG**AAAGATGCATTTACTTCCCAATGGA
*Tn-ago2*_T7_Rv	**TAATACGACTCACTATAGGG**CCAAGATTAACGAACTTTGGTTTGG

<u>Table S2</u>: Primer sequences used for qRT-PCR.

*Tn-rps18*_qPCR_Fw	GCTCTGTATCGCCGTAACCA
*Tn-rps18*_qPCR_Rv	TGCCGTTTCGAACACAAGAC
*Tn-ef1a*_qPCR_Fw	GTCCACAACCACTGGTCACT
*Tn-ef1a*_qPCR_Rv	TCACGCTCAGCCTTCAGTTT
*Tn-actin*_qPCR_Fw	CGCACACGGTGCCCATCTA
*Tn-actin*_qPCR_Rv	CTCGGTGAGGATCTTCATCA
*Tn-tubulin*_qPCR_Fw	CGCAACCTCGACATTGAAC
*Tn-tubulin*_qPCR_Rv	TCGGTAAGGTCGACGTTGA
*Tn-elf4a*_qPCR_Fw	GTGAGCGCGAAGTTATTATGC
*Tn-elf4a*_qPCR_Rv	AGAAACTTGCTGCACGTCAAT
*Tn-dcr2*_qPCR_Fw	CGGTGACTTGCAGACTGTCT
*Tn-dcr2*_qPCR_Rv	TTCACTGGCTGTTGCGGTAT
*Tn-ago2*_qPCR_Fw	GACTGCGCGTCGTATAAACC
*Tn-ago2*_qPCR_Rv	TGACATTGCCGTCCCTCAAA
*mlv*_qPCR_Fw	TGAGTTGCGTTCCGAATGGA
*mlv*_qPCR_Rv	ACCAAGACGGGTGCTGATTT

<u>Table S3</u>: Primer sequences used for detection of *Bm*-MLV in High Five cells.

mlv-Fw1	CGTCCTCCCAACTTGTTTGT
mlv-Rv1	CTCCATTTTCTTCCGTTCCA
mlv-Fw2	ATTCTTCGGCGTCTCGACTA
mlv-Rv2	AGTTGAAGGGTTGGTCGTTG
